# Associations between physical activity and sleep quality among college students: the chain-mediating roles of rumination and emotion regulation strategies

**DOI:** 10.3389/fpsyg.2026.1835863

**Published:** 2026-05-18

**Authors:** Yingying Gu, Rui Li, Jianqin Chen, Jiameng Sun, Mohamad Nizam Nazarudin, Wanning Zhu

**Affiliations:** 1 Faculty of Education, Universiti Kebangsaan Malaysia, Bangi, Selangor, Malaysia; 2 Institute of Sports Science, Nantong University, Nantong, China; 3 Education School, Quanzhou Vocational and Technical University, Quanzhou, China; 4 Sports Institute, Chengdu University of Technology, Chengdu, China

**Keywords:** college students, emotion regulation, physical activity, rumination, sleep quality

## Abstract

**Objective:**

Sleep problems are common among college students and represent an important public health concern. Previous studies have shown that physical activity is related to sleep quality. However, the psychological processes involved in this association are not fully understood. This study examined the association between physical activity level and sleep quality in college students and explored the sequential roles of rumination and emotion regulation strategies, including cognitive reappraisal and expressive suppression.

**Methods:**

This study adopted a cross-sectional design. Data were collected from college students using self-report questionnaires measuring physical activity, rumination, emotion regulation strategies, and sleep quality. Correlation analysis and structural equation modeling were conducted. Bootstrapping was used to test the proposed chain mediation model.

**Results:**

Physical activity level was significantly associated with sleep quality. Rumination significantly mediated this association. In addition, two sequential indirect pathways were significant: physical activity → rumination → cognitive reappraisal → sleep quality (effect = −0.003, 95% CI [−0.006, −0.001]) and physical activity → rumination → expressive suppression → sleep quality (effect = −0.003, 95% CI [−0.006, −0.001]). The total indirect effect was also significant (effect = −0.029, 95% CI [−0.035, −0.023]).

**Conclusion:**

This study provides an integrated view of how physical activity, rumination, and emotion regulation strategies are associated with sleep quality among college students. The findings highlight the importance of considering cognitive and emotional factors when examining sleep problems in this population. These results may inform physical activity–based approaches to sleep and mental health promotion in university settings. Due to the cross-sectional design, future studies using longitudinal or intervention methods are needed to further examine these associations.

## Introduction

Sleep problems are common among college students and have become a widespread concern during the university years (Schlarb et al., 2017). Academic demands, social relationships, and uncertainty about future development often make it difficult for students to fully relax at night. Even after going to bed, many students continue to think about daily events or worry about unfinished tasks. As a result, they may experience difficulty falling asleep, shallow sleep, or frequent awakenings during the night. Poor sleep quality over time can impair next-day learning efficiency and attention. It may also increase emotional instability and elevate the risk of depressive and anxiety symptoms, thereby contributing to a negative cycle that undermines both mental and physical health (Ghoul et al., 2025). Therefore, examining the factors associated with sleep quality in the daily lives of college students is of clear practical importance.

In previous research, physical activity has been widely regarded as an important behavioral factor associated with better sleep quality (Alnawwar et al., 2023). Compared with pharmacological approaches or professional psychological treatment, physical activity is low in cost, involves fewer risks, and is easier to promote in campus settings. For these reasons, it has received sustained attention in the fields of health promotion and public health (Matos Fialho et al., 2025). A large body of research has shown that individuals with higher levels of physical activity tend to report better sleep outcomes, such as shorter sleep onset latency, fewer nighttime awakenings, and more positive overall sleep experiences (Kredlow et al., 2015). However, some studies have reported that the association between physical activity and sleep quality is not equally strong across different populations (Dolezal et al., 2017). This finding suggests that physical activity may not influence sleep quality through a single physiological fatigue mechanism alone. Instead, the association may be shaped by individual differences in psychological states and cognitive processing. In recent years, an increasing number of studies have begun to interpret this relationship from cognitive and emotional perspectives. These studies emphasize that physical activity may be related to sleep outcomes through its associations with thinking patterns and emotion regulation processes (Gerber et al., 2025).

From the perspective of daily experience, factors affecting sleep among college students often extend beyond the physical level and are more closely related to psychological processes that are difficult to disengage from. Repeatedly thinking about academic performance, interpersonal interactions, or future plans before bedtime is a common experience among college students (Pillai and Drake, 2015; Dass and Pandey, 2025). This pattern of thinking, characterized by persistent and repetitive focus on negative content, is referred to as rumination and has been identified as an important cognitive risk factor for sleep disturbance (Nolen-Hoeksema et al., 2008; Watkins and Roberts, 2020). Rumination tends to maintain a heightened level of psychological arousal, making it difficult for individuals to relax even in a quiet environment. As a result, it may delay sleep onset or reduce overall sleep quality (Kamene, 2025). Previous research has shown that rumination is closely associated with psychological problems such as depression and anxiety. It has also been identified as a key mediating factor in the associations between physical activity and various psychological and behavioral outcomes, including subjective well-being and internet addiction (Doğan, 2024; Wang et al., 2025a; Wang et al., 2025c). In contrast, physical activity may help individuals temporarily disengage from repetitive negative thinking by shifting attention, reducing stress, and enhancing cognitive control. These processes may create more favorable psychological conditions for sleep at night (Smith and Merwin, 2021; Wang et al., 2025b). Therefore, rumination is likely to play a mediating role in the association between physical activity and sleep quality.

Beyond rumination, the ways in which individuals regulate their emotions may also be associated with sleep outcomes (Palmer and Alfano, 2017; Vandekerckhove and Wang, 2017). Emotion regulation is not a single ability but includes a range of strategies. Among these, cognitive reappraisal and expressive suppression are the two most commonly studied strategies (Gross, 1998; Cutuli, 2014). Cognitive reappraisal involves changing the way one interprets or understands the meaning of a situation in order to reduce emotional responses. It is generally considered a more adaptive strategy and may help lessen the prolonged impact of negative emotions (Clark, 2022). In contrast, expressive suppression refers to the deliberate inhibition of emotional expression after emotions have already arisen. Although this strategy may reduce outward emotional expression in the short term, it is often associated with greater psychological burden (Caramanica et al., 2023). Previous studies have shown that individuals who tend to use cognitive reappraisal report better sleep experiences, whereas those who frequently rely on expressive suppression are more likely to report sleep problems (Ballot et al., 2021; Cox et al., 2016; Li et al., 2023).

Further evidence suggests that rumination and emotion regulation strategies are not independent processes but may interact with and reinforce each other. Persistent rumination tends to consume substantial cognitive resources. This makes it more difficult for individuals to interpret emotional events in a flexible and multi-perspective manner, thereby reducing the likelihood of using adaptive strategies such as cognitive reappraisal (Battista et al., 2023; Hu et al., 2024). At the same time, rumination may increase reliance on expressive suppression as a way of managing negative emotions, which can further intensify internal psychological burden (Zou et al., 2024). Previous studies have observed similar sequential patterns across different psychological outcomes. These findings suggest that physical activity may be associated with lower levels of rumination, which in turn create conditions that support more adaptive regulatory processes and are related to subsequent psychological and behavioral outcomes (Zhou and Wang, 2025). This process may be particularly salient at night. When external stimulation is reduced, rumination and maladaptive emotion regulation strategies are more likely to be amplified, thereby exerting a negative influence on sleep quality (Palmer and Alfano, 2017; You et al., 2023). From this perspective, physical activity may not only be associated with reduced rumination but may also relate to sleep outcomes indirectly through its associations with emotion regulation strategies.

Based on the theoretical and empirical background outlined above, this study focused on college students and examined the association between physical activity and sleep quality. It further explored the chain-mediating roles of rumination and two emotion regulation strategies, namely cognitive reappraisal and expressive suppression. By adopting an integrated perspective that considers both cognitive processes and emotion regulation, this study aimed to clarify the psychological pathways linking physical activity and sleep quality among college students. The findings are expected to provide more targeted theoretical insights and practical implications for physical activity–based sleep and mental health promotion in university settings.

## Research hypotheses

Based on the theoretical framework outlined above and the daily context of college students, this study proposes that physical activity may be associated with sleep quality through its relationships with cognitive processing and emotion regulation strategies. Specifically, physical activity may be directly associated with sleep quality. It may also be indirectly associated with sleep quality by relating to lower levels of rumination and, in turn, different patterns of emotion regulation. These associations may form a sequential psychological pathway linking physical activity and sleep quality. Accordingly, the following hypotheses were proposed.

*H1*: Physical activity level is significantly associated with sleep quality among college students.

*H2*: Physical activity level is negatively associated with rumination.

*H3*: Rumination is positively associated with poorer sleep quality.

*H4*: Rumination mediates the association between physical activity level and sleep quality.

*H5-1*: Physical activity level is positively associated with cognitive reappraisal and negatively associated with expressive suppression.

*H5-2*: Rumination is negatively associated with cognitive reappraisal and positively associated with expressive suppression.

*H6*: Cognitive reappraisal is associated with better sleep quality, whereas expressive suppression is associated with poorer sleep quality.

*H7*: Rumination and emotion regulation strategies (cognitive reappraisal and expressive suppression) sequentially mediate the association between physical activity level and sleep quality.

## Materials and methods

### Ethics approval and informed consent

This study was reviewed and approved by the Ethics Committee of Nantong University (Ethical Approval No.: TD-2024-109). Prior to data collection, all participants were informed about the purpose of the study, the study procedures, and relevant considerations. Electronic informed consent was obtained from all participants before they completed the questionnaire. Participation was entirely voluntary, and participants were informed that they could withdraw from the study at any time without any consequences. This study involved human participants, and all procedures were conducted in accordance with institutional ethical standards and the Declaration of Helsinki.

### Data source and participants

The data were collected from undergraduate students at four universities located in Jiangsu and Zhejiang provinces, China. A cluster sampling method was used, with schools or classes serving as the sampling units. Data collection was conducted in coordination with the participating universities, and eligible clusters were invited to complete the questionnaire survey. Data were collected using an online questionnaire created on the Wenjuanxing^1^ platform. The survey was administered in a classroom setting, where participants completed the questionnaire collectively under the supervision of trained researchers. Standardized instructions were provided to ensure consistency in the testing environment and administration procedures. The survey was conducted between September and November 2025. A total of 950 questionnaires were returned. The exclusion criteria included: (1) incomplete questionnaires; (2) questionnaires showing clearly invalid response patterns (e.g., extremely short completion time); and (3) cases with substantial missing data on key variables. After data screening, 937 valid responses were retained for analysis. The final sample included 437 male and 500 female students, with a mean age of 20.32 years (SD = 1.59). The survey did not include a separate screening item regarding prior or current clinically diagnosed sleep disorders. Accordingly, participants with such conditions were not excluded on that basis, and this variable was not included as a covariate in the present analyses.

### Measures

#### Physical activity

Physical activity was assessed using the Physical Activity Rating Scale–3 (PARS-3), developed by Liang D. C. and widely used in Chinese populations (Liang, 1994). The scale has demonstrated good reliability and validity in previous studies. The PARS-3 assesses physical activity across three dimensions: exercise frequency, exercise duration, and exercise intensity. It consists of three items, each rated on a 5-point Likert scale ranging from 1 to 5. The total physical activity score was calculated using the following formula: Physical activity score = exercise frequency × (exercise duration − 1) × exercise intensity, with a possible score range of 0 to 100. Based on the total score, physical activity levels were categorized into three groups: low (≤ 19), moderate (20–42), and high (≥ 43). The PARS-3 captures the overall level of physical activity through frequency, duration, and intensity; however, it does not assess the specific type or timing of physical activity. Therefore, the present study was not able to examine whether different exercise modalities or evening physical activity were differentially associated with sleep quality.

#### Rumination

Rumination was assessed using the Ruminative Responses Scale (RRS). The scale was originally developed by Nolen-Hoeksema and later revised into a Chinese version by Han and Yang (2009). The RRS consists of 22 items and is rated on a 4-point Likert scale, with higher scores indicating a stronger tendency toward rumination. The scale comprises three dimensions: symptom rumination, brooding, and reflective pondering. Symptom rumination includes Items 1, 2, 3, 4, 6, 8, 9, 14, 17, 18, 19, and 22; brooding includes Items 5, 10, 13, 15, and 16; and reflective pondering includes Items 7, 11, 12, 20, and 21. Previous studies have shown that the Chinese version of the RRS has been widely used and validated among Chinese college students, demonstrating good structural validity and reliability (Wang et al., 2025a). For descriptive and correlational analyses, the RRS total score and subscale scores were computed as mean item scores rather than summed scores. In the present study, the RRS showed high internal consistency, with a Cronbach’s alpha coefficient of 0.937.

#### Sleep quality

Sleep quality was assessed using the Pittsburgh Sleep Quality Index (PSQI). The PSQI was developed by Buysse and colleagues and is widely used to evaluate sleep quality over the past month. In the present study, the Chinese version translated by Liu and colleagues was used (Liu et al., 1996). The PSQI consists of 19 self-rated items and 5 observer-rated items, of which 18 self-rated items are included in scoring. These items yield seven component scores: subjective sleep quality (1 item), sleep latency (2 items), sleep duration (1 item), habitual sleep efficiency (3 items), sleep disturbances (9 items), use of sleep medication (1 item), and daytime dysfunction (2 items). Each component is rated on a 4-point scale ranging from 0 to 3, and the global PSQI score ranges from 0 to 21, with higher scores indicating poorer sleep quality. In the present study, PSQI scores were analyzed as a continuous variable rather than being used to classify participants into sleep-quality categories.

#### Emotion regulation strategies

Emotion regulation strategies were assessed using the Emotion Regulation Questionnaire (ERQ), developed by Gross and John (2003). The Chinese version of the ERQ was revised by Wang et al. (2007) and has been widely used among Chinese college students, demonstrating good reliability and validity. The ERQ consists of two dimensions: cognitive reappraisal and expressive suppression, each comprising seven items. The cognitive reappraisal dimension assesses the tendency to regulate emotions by changing how one interprets or understands emotion-eliciting situations. The expressive suppression dimension measures the tendency to inhibit the outward expression of emotions after they have been generated. All items are rated on a 7-point Likert scale, with higher scores indicating more frequent use of the corresponding emotion regulation strategy. For consistency with the RRS, ERQ dimension scores were also calculated as mean item scores. In the present study, the Cronbach’s alpha coefficients were 0.923 for cognitive reappraisal and 0.925 for expressive suppression, indicating high internal consistency.

### Statistical analysis

Statistical analyses were conducted using SPSS 26.0 and AMOS 26.0. First, descriptive statistics were calculated for all study variables, including means, standard deviations, skewness, and kurtosis, to examine data distribution characteristics and normality. Harman’s single-factor test was then performed to assess the potential influence of common method bias. For measurement model evaluation, confirmatory factor analysis (CFA) was conducted using AMOS to examine the structural validity of the latent constructs. Model fit was assessed using multiple indices, including the chi-square to degrees of freedom ratio (*x*^2^/df), root mean square error of approximation (RMSEA), comparative fit index (CFI), Tucker–Lewis index (TLI), goodness-of-fit index (GFI), incremental fit index (IFI), and root mean square residual (RMR). Composite reliability (CR) and average variance extracted (AVE) were calculated to assess convergent validity, and discriminant validity was evaluated using the Fornell–Larcker criterion. In addition, competing measurement models with different factor structures were compared to further examine the adequacy of the proposed measurement model. In the preliminary analysis, independent-samples *t*-tests and correlation analyses were conducted to examine differences and associations among the main study variables across demographic characteristics, such as sex and age. Subsequently, mediation analyses were performed to test the chain-mediating roles of rumination and emotion regulation strategies in the association between physical activity and sleep quality. Bootstrapping procedures with 5,000 resamples were used to estimate confidence intervals for indirect effects. An indirect effect was considered significant when the 95% confidence interval did not include zero. Although the RRS includes three subdimensions (symptom rumination, reflective pondering, and brooding), the present study focused on the total rumination score in the main analyses to capture the general tendency toward repetitive negative thinking and to maintain model parsimony. Subscale-level analyses were conducted as supplementary analyses and are reported in Supplementary material.

## Results

### Common method bias test

Given that all data were collected using self-report questionnaires, Harman’s single-factor test was conducted to examine the potential impact of common method bias. The results showed that the first unrotated factor accounted for 11.659% of the total variance, which did not exceed the critical threshold of 40% (Podsakoff et al., 2003). This finding suggests that common method bias was not a serious concern in the present study, and its influence on the study results was within an acceptable range.

### Descriptive statistics and correlation analysis

#### Descriptive statistics

Descriptive statistics for the main study variables are presented in Table 1. The means, standard deviations, skewness, and kurtosis values of all variables were within acceptable ranges (Kline, 2023), indicating that the data distributions were generally appropriate and met the assumptions for subsequent parametric analyses.

**TABLE 1:** Descriptive statistics of the study variables.

Variable	Score type	Range	Mean	SD	Skewness	Kurtosis
Rumination (Total)	Mean score	1–4	2.351	0.728	0.071	−0.896
–Symptom rumination	Mean score	1–4	2.406	0.836	0.003	−1.109
–Reflective pondering	Mean score	1–4	2.250	0.832	0.339	−0.795
–Brooding	Mean score	1–4	2.321	0.854	0.205	−0.912
Cognitive reappraisal	Mean score	1–7	4.664	1.362	−0.466	−0.558
Expressive suppression	Mean score	1–7	3.333	1.366	0.481	−0.501
Physical activity level	Total score	0–100	29.259	30.093	0.928	−0.262
Sleep quality(PSQI)	Total score	0–21	6.335	4.606	0.937	0.252

SD, standard deviation. Higher PSQI scores indicate poorer sleep quality. Physical activity and sleep quality are reported as total scores. Rumination and emotion regulation variables are reported as mean item scores to facilitate comparison across dimensions.

Specifically, overall rumination among college students was at a moderate level, with similar mean scores across the three dimensions of symptom rumination, reflective pondering, and brooding. Regarding emotion regulation strategies, the mean level of cognitive reappraisal was higher than that of expressive suppression, suggesting that college students tended to rely more on cognitive reappraisal when regulating their emotions. Physical activity levels showed substantial individual variability within the sample. In addition, scores on the PSQI were at a moderately low level, indicating that there remained room for improvement in overall sleep quality among the participants.

#### Correlation analysis

Based on this, correlation analyses were conducted to examine the relationships among the study variables, and the results are presented in Table 2. The correlation analysis showed that physical activity level was significantly negatively correlated with sleep quality (*r* = −0.372, *p* < 0.001), indicating that higher levels of physical activity were associated with fewer sleep problems. Physical activity level was also significantly negatively correlated with rumination (*r* = −0.327, *p* < 0.001) and expressive suppression (*r* = −0.317, *p* < 0.001), and significantly positively correlated with cognitive reappraisal (*r* = 0.302, *p* < 0.001).

**TABLE 2:** Means, standard deviations, and correlations among study variables.

Variable	M ± SD	1	2	3	4	5	6	7
1. Sex	1.53 ± 0.50	1	1	1	√AVE = 0.806	√AVE = 0.746	√AVE = 0.750	1
2. Age	20.32 ± 1.59	0.018						
3. Physical activity level	29.26 ± 30.09	0.032	−0.003					
4. Rumination	2.35 ± 0.73	−0.007	−0.022	−0.327**				
5. Cognitive reappraisal	4.66 ± 1.36	0.003	−0.013	0.302**	−0.434**			
6. Expressive suppression	3.33 ± 1.37	−0.016	−0.008	−0.317**	0.352**	−0.641**		
7. Sleep quality (PSQI)	6.34 ± 4.61	0.010	−0.017	−0.372**	0.425**	−0.407**	0.392**	

Note. Values on the diagonal (in bold) represent the square roots of the average variance extracted (AVE). *p* < 0.05,

** *p* < 0.01 (two-tailed). Higher PSQI scores indicate poorer sleep quality.

Rumination was significantly positively correlated with sleep quality (*r* = 0.425, *p* < 0.001), such that higher levels of rumination were associated with poorer sleep quality. In addition, rumination was significantly negatively correlated with cognitive reappraisal (*r* = −0.434, *p* < 0.001) and significantly positively correlated with expressive suppression (*r* = 0.352, *p* < 0.001). With regard to emotion regulation strategies, cognitive reappraisal was significantly negatively correlated with sleep quality (*r* = −0.407, *p* < 0.001), whereas expressive suppression was significantly positively correlated with sleep quality (*r* = 0.392, *p* < 0.001). Additional correlation analyses involving the three rumination subdimensions are presented in Supplementary material, showing a pattern of associations consistent with those observed for the total rumination score.

In addition, the correlation coefficients among the latent variables were all lower than the square roots of their corresponding average variance extracted (AVE) values, satisfying the Fornell–Larcker criterion. This finding further supports the discriminant validity of the study variables (Kline, 2023). Overall, the directions of the correlations were consistent with the proposed hypotheses, providing preliminary support for the subsequent mediation and chain mediation analyses.

### Measurement model testing

#### Confirmatory factor analysis and model fit

To examine the structural validity of the study variables, confirmatory factor analysis (CFA) was conducted using AMOS. The results indicated that the five-factor model, including rumination, cognitive reappraisal, expressive suppression, physical activity level, and sleep quality, demonstrated a good overall fit to the data, with all fit indices meeting the recommended criteria. Specifically, the chi-square to degrees of freedom ratio (*x*^2^/df) was 1.465, the root mean square error of approximation (RMSEA) was 0.022, the comparative fit index (CFI) was 0.987, the Tucker–Lewis index (TLI) was 0.985, and the root mean square residual (RMR) was 0.058. These results indicate that the measurement model fit the sample data well (Fornell and Larcker, 1981).

To further evaluate the adequacy of the measurement model, several competing models were constructed and compared, as shown in Table 3. Compared with the two-factor, three-factor, four-factor, and one-factor models, the five-factor model showed clear advantages across key fit indices, including *x*^2^/df, RMSEA, CFI, and TLI, and demonstrated a substantially better overall fit than the alternative models. These findings suggest that the proposed five-factor measurement model exhibits good discriminant validity and structural adequacy.

**TABLE 3:** Fit indices of competing measurement models.

Model	*x*^2^/df	RMSEA	CFI	TLI	RMR
Second-order model (2 factors: Rumination + Emotion Regulation)	1.468	0.022	0.986	0.985	0.061
Second-order model (3 factors: Rumination + Cognitive Reappraisal + Expressive Suppression)	1.486	0.022	0.986	0.985	0.096
Five-factor model (full model)	1.465	0.022	0.987	0.985	0.058
Four-factor model (Symptom Rumination + Reflective Pondering + Brooding + Cognitive Reappraisal/Expressive Suppression)	3.180	0.148	0.936	0.932	0.109
Three-factor model (Symptom Rumination/Reflective Pondering/Brooding + Cognitive Reappraisal + Expressive Suppression)	5.190	0.067	0.877	0.869	0.084
Two-factor model (Symptom Rumination/Reflective Pondering/Brooding + Cognitive Reappraisal/Expressive Suppression)	6.882	0.079	0.827	0.816	0.125
One-factor model (all indicators loaded on a single factor)	14.282	0.361	0.609	0.585	0.361

#### Convergent validity

Based on the satisfactory fit of the measurement model, convergent validity was further examined for each latent construct, and the results are presented in Table 4. All standardized factor loadings were statistically significant and exceeded 0.70. In addition, the composite reliability (CR) values for all constructs were above 0.80, and the average variance extracted (AVE) values exceeded the recommended threshold of 0.50. These results indicate that the measurement model demonstrated good convergent validity.

**TABLE 4:** Convergent validity of the measurement model.

Latent construct	Indicator	Standardized factor loading	CR	AVE
Rumination	Symptom rumination	0.861	0.847	0.650
	Reflective pondering	0.702		
	Brooding	0.846		
Emotion regulation	Cognitive reappraisal	0.934	0.840	0.727
	Expressive suppression	0.762		

CR, composite reliability; AVE, average variance extracted. All standardized factor loadings were statistically significant (*p* < 0.001).

#### Discriminant validity

As shown in Table 5, the square roots of the average variance extracted (AVE) for all latent constructs were greater than the corresponding inter-construct correlation coefficients, satisfying the Fornell–Larcker criterion. These results support the discriminant validity of the measurement model.

**TABLE 5:** Discriminant validity of the measurement model.

Construct	1	2
1. Rumination	0.806	0.852
2. Emotion regulation	−0.525**	

*p < 0.05,

**p < 0.01.

Overall, the measurement model demonstrated adequate structural validity, convergent validity, and discriminant validity. This provides a sound measurement foundation for subsequent structural model analyses.

### Demographic differences

#### Sex differences in study variables

To examine sex differences in the study variables, independent-samples *t*-tests were conducted with sex as the grouping variable. Differences in rumination (symptom rumination, reflective pondering, and brooding), emotion regulation strategies (cognitive reappraisal and expressive suppression), physical activity level, and sleep quality were examined. The results are presented in Table 6.

**TABLE 6:** Sex differences in study variables.

Variable	Male (M ± SD)	Female (M ± SD)	*t*	*p*
Rumination	2.36 ± 0.72	2.35 ± 0.74	0.206	0.837
Symptom rumination	2.42 ± 0.82	2.39 ± 0.85	0.467	0.641
Reflective pondering	2.25 ± 0.82	2.25 ± 0.84	0.052	0.958
Brooding	2.31 ± 0.82	2.33 ± 0.89	−0.377	0.707
Emotion regulation	4.01 ± 0.62	3.99 ± 0.54	0.476	0.634
Cognitive reappraisal	4.66 ± 1.34	4.67 ± 1.38	−0.087	0.931
Expressive suppression	3.36 ± 1.34	3.31 ± 1.39	0.489	0.625
Physical activity level	28.22 ± 28.68	30.17 ± 31.28	−0.997	0.319
Sleep quality(PSQI)	6.28 ± 4.62	6.38 ± 4.60	−0.319	0.750

Higher PSQI scores indicate poorer sleep quality.

The results indicated that there were no significant sex differences in any of the study variables, including rumination, emotion regulation strategies, physical activity level, and sleep quality (all *p*> 0.05). These findings suggest that, within the present sample, sex was not significantly associated with the main study variables.

#### Associations between age and study variables

Age was treated as a continuous variable, and correlation analyses were conducted to examine the associations between age and the study variables. The results are presented in Table 7.

**TABLE 7:** Age differences in study variables.

Variable	Age
Rumination	−0.022
Symptom rumination	−0.024
Reflective pondering	0.028
Brooding	−0.053
Emotion regulation	−0.025
Cognitive reappraisal	−0.013
Expressive suppression	−0.008
Physical activity level	−0.003
Sleep quality(PSQI)	−0.017

The analysis showed that age was not significantly correlated with any of the study variables, including the dimensions of rumination, emotion regulation strategies, physical activity level, and sleep quality (all *p* > 0.05). These findings suggest that, within the age range of the present college student sample, age was not meaningfully associated with the main study variables.

### Chain mediation analysis

To further examine the mediating roles of rumination and emotion regulation strategies in the association between physical activity and sleep quality, chain mediation models were tested using hierarchical regression analyses combined with the bootstrap method. Two mediation pathways were specified, with rumination and cognitive reappraisal, as well as rumination and expressive suppression, entered sequentially as mediators. The results of the regression analyses are presented in Tables 8, 9, and the bootstrap mediation results are shown in Table 10.

**TABLE 8:** Chain mediation analysis with rumination and cognitive reappraisal as mediators.

Variable type	Variable	Model1	Model 2	Model 3	Model 4
		X-M1	X-M2	X-Y	X/M1/M2-Y
Constant	Constant	2.791**	6.456**	8.794**	7.849**
Control variables	Sex	0.006	−0.014	0.211	0.186
	Age	−0.011	−0.017	−0.055	−0.045
Independent variable	Physical activity level	−0.008**	0.008**	−0.057**	−0.034**
Mediators	Rumination	–	−0.704**	–	1.597**
	Cognitive reappraisal	–	–	–	−0.782**
*R* ^2^		0.107	0.218	0.139	0.284
Adjusted *R*^2^		0.104	0.214	0.136	0.280
△*R*^2^		0.107	0.218	0.139	0.145
*F*		37.352**	64.868**	50.318**	73.860**

X, Physical activity level; M1, Rumination; M2, Cognitive reappraisal; Y, Sleep quality.

*p < 0.05,

**p < 0.01.

**TABLE 9:** Chain mediation analysis with rumination and expressive suppression as mediators.

Variable type	Variable	Model1	Model 2	Model 3	Model 4
		X-M1	X-M3	X-Y	X/M1/M3-Y
Constant	Constant	2.791**	2.483**	8.794**	0.892
Control variables	Sex	0.006	−0.019	0.211	0.212
	Age	−0.011	−0.002	−0.055	−0.030
Independent variable	Physical activity level	−0.008**	−0.010**	−0.057**	−0.032**
Mediators	Rumination	–	0.522**	–	1.746**
	Expressive suppression	–	–	–	0.769**
*R* ^2^		0.107	0.170	0.139	0.285
Adjusted *R*^2^		0.104	0.166	0.136	0.281
△*R*^2^		0.107	0.170	0.139	0.146
*F*		37.352**	47.585**	50.318**	74.339**

X, Physical activity level; M1, Rumination; M2, Cognitive reappraisal; Y, Sleep quality.

*p < 0.05,

**p < 0.01.

**TABLE 10:** Direct, indirect, and chain mediation effects.

Type of effect	Path	Effect	95% CI lower	95% CI upper	*p*	Effect ratio
Direct effect	Physical activity level → Sleep quality	−0.028	−0.037	−0.020	< 0.001	49.12%
	Rumination → Sleep quality	1.845	1.243	2.455	0.001	–
	Cognitive reappraisal → Sleep quality	−0.470	−0.800	−0.119	0.007	–
	Expressive suppression → Sleep quality	0.512	0.212	0.821	0.001	–
	Physical activity level → Rumination	−0.008	−0.010	−0.006	< 0.001	–
	Physical activity level → Cognitive reappraisal	0.006	0.003	0.009	<0.001	–
	Physical activity level → Expressive suppression	−0.008	−0.011	−0.005	< 0.001	–
	Rumination → Cognitive reappraisal	−0.893	−1.112	−0.708	<0.001	–
	Rumination → Expressive suppression	0.738	0.547	0.974	< 0.001	–
Simple indirect effects	Physical activity → Rumination → Sleep quality	−0.015	−0.022	−0.009	< 0.001	26.32%
	Physical activity → Cognitive reappraisal → Sleep quality	−0.003	−0.006	−0.001	0.003	5.26%
	Physical activity → Expressive suppression → Sleep quality	−0.004	−0.008	−0.002	0.001	7.02%
Chain indirect effects	Physical activity → Rumination → Cognitive reappraisal → Sleep quality	−0.003	−0.006	−0.001	0.005	5.26%
	Physical activity → Rumination → Expressive suppression → Sleep quality	−0.003	−0.006	−0.001	0.001	5.26%
Total indirect effect	Physical activity →…→ Sleep quality	−0.029	−0.035	−0.023	< 0.001	50.88%
Total effect	Physical activity →…→ Sleep quality	−0.057	−0.066	−0.048	< 0.001	–

Sleep quality was measured using the Pittsburgh Sleep Quality Index, with higher scores indicating poorer sleep quality. Bootstrap confidence intervals were based on 5,000 resamples. A mediation effect was considered significant when the 95% confidence interval did not include zero.

#### Regression analyses of the chain mediation models

After controlling for sex and age, the association between physical activity level and rumination was first examined. The results indicated that physical activity level was significantly negatively associated with rumination (*p* < 0.01), suggesting that higher levels of physical activity were associated with lower levels of rumination among college students. Emotion regulation strategies were then included in the mediation models. Specifically, rumination was entered as a predictor of emotion regulation strategies. In the model with cognitive reappraisal as the second mediator (see Table 8), rumination was significantly negatively associated with cognitive reappraisal (*p* < 0.01), indicating that higher levels of rumination were related to lower use of cognitive reappraisal. In turn, cognitive reappraisal was significantly negatively associated with sleep quality (*p* < 0.01).

In the model with expressive suppression as the second mediator (see Table 9), rumination was entered as a predictor of expressive suppression. The results showed that rumination was significantly positively associated with expressive suppression (*p* < 0.01), indicating that higher levels of rumination were related to greater use of expressive suppression. Expressive suppression was, in turn, significantly positively associated with sleep quality (*p* < 0.01). After simultaneously including rumination and emotion regulation strategies in the model, the direct association between physical activity level and sleep quality remained significant (*p* < 0.01), indicating the presence of a partial mediation effect between physical activity and sleep quality.

#### Bootstrap tests of chain mediation effects

To further examine the significance of the mediation effects, a bootstrap procedure with 5,000 resamples was conducted. Bias-corrected 95% confidence intervals were estimated for all direct, indirect, and chain-mediated paths. As shown in Table 10, mediation effects were considered statistically significant when the corresponding confidence intervals did not include zero.

Bootstrap analyses showed that the indirect effects of physical activity on sleep quality were significant. Specifically, the indirect effect through rumination was significant (effect = −0.015, 95% CI [−0.022, −0.009], *p* < 0.001). The indirect effect through cognitive reappraisal was also significant (effect = −0.003, 95% CI [−0.006, −0.001], *p* = 0.003), as was the indirect effect through expressive suppression (effect = −0.004, 95% CI [−0.008, −0.002], *p* = 0.001). Importantly, the specific chain indirect effect through rumination and cognitive reappraisal was significant (effect = −0.003, 95% CI [−0.006, −0.001], *p* = 0.005). Similarly, the chain indirect effect through rumination and expressive suppression was also significant (effect = −0.003, 95% CI [−0.006, −0.001], *p* = 0.001). The total indirect effect was significant (effect = −0.029, 95% CI [−0.035, −0.023], *p* < 0.001), accounting for 50.88% of the total effect. These findings indicate that the association between physical activity and sleep quality is partially mediated by rumination and emotion regulation strategies, including both simple and sequential pathways.

## Discussion

### Main findings

This study examined the associations between physical activity level and sleep quality among college students and further explored the chain associations involving rumination and two emotion regulation strategies, cognitive reappraisal and expressive suppression. The results indicate that physical activity, rumination, emotion regulation strategies, and sleep quality are closely related rather than operating independently. Together, these variables form a connected psychological and behavioral pattern that helps explain individual differences in sleep quality among college students.

First, physical activity level was significantly associated with sleep quality. College students who reported higher levels of physical activity tended to experience fewer sleep problems. This finding is consistent with previous studies showing a robust relationship between physical activity and sleep health (Kredlow et al., 2015; Ghrouz et al., 2019). From a behavioral perspective, higher physical activity levels are often linked to more regular daily routines and more stable sleep–wake patterns. In addition, physical activity may help regulate physiological arousal and recovery processes, which can make it easier to fall asleep and reduce subjective sleep-related discomfort (Kredlow et al., 2015). It should be emphasized that, because of the cross-sectional design, these findings reflect associations rather than causal effects of physical activity on sleep quality.

Second, the results support a model in which rumination plays an important role in the association between physical activity level and sleep quality. Physical activity level was negatively associated with rumination, whereas higher levels of rumination were related to poorer sleep quality. This pattern suggests that the link between physical activity and sleep quality may partly depend on how individuals process their daily experiences at a cognitive level. Rumination involves repetitive and negative thinking and is particularly likely to occur before bedtime (Pillai and Drake, 2015). During this period, reduced external stimulation may allow unresolved concerns to occupy attention, maintain cognitive and emotional arousal, and interfere with relaxation, thereby delaying sleep onset (Arbinaga et al., 2019). In this sense, rumination appears to be a key cognitive process connecting daytime behaviors with nighttime sleep experiences, while other pathways may also exist.

Further analyses showed that emotion regulation strategies were differently associated with rumination and sleep quality. Higher levels of rumination were associated with lower use of cognitive reappraisal, and students who used cognitive reappraisal more frequently generally reported better sleep quality. Cognitive reappraisal may help individuals reinterpret stressful events and reduce the lasting impact of negative emotions, which can ease cognitive and emotional activation before sleep (Vandekerckhove and Wang, 2017; Xu et al., 2025). In contrast, rumination was positively associated with expressive suppression, and greater reliance on expressive suppression was linked to more severe sleep problems. Suppressing emotional expression may leave emotional experiences unresolved, increasing internal tension and psychological burden at night and, in turn, worsening sleep disturbance (Wang et al., 2022). These findings do not suggest that emotion regulation strategies are inherently adaptive or maladaptive. Instead, their associations with sleep quality appear to depend on the broader cognitive context, particularly the level of rumination.

Taken together, the findings support an integrated framework in which physical activity level, rumination, emotion regulation strategies, and sleep quality interact with one another. Focusing only on physiological or behavioral factors may be insufficient for understanding sleep problems among college students. Considering cognitive processing patterns and emotion regulation strategies provides a more comprehensive view of why sleep quality varies across individuals.

### Theoretical implications

This study provides an integrated theoretical examination of the relationships among physical activity, rumination, emotion regulation strategies, and sleep quality, offering a combined cognitive–emotional perspective for understanding sleep problems among college students. Compared with previous studies that have largely focused on single variables or bivariate associations (Wang et al., 2025c), this study constructed a chain mediation model to examine the association structure among behavioral, cognitive, and emotional factors within a unified analytical framework. In doing so, it extends the theoretical pathways through which physical activity is related to sleep quality.

First, this study introduces rumination as a key cognitive variable into research on physical activity and sleep quality, supplementing prior approaches that primarily emphasized physiological mechanisms or behavioral frequency. Existing theories suggest that sleep problems are not only related to levels of physiological arousal but are also closely linked to cognitive processing during the pre-sleep period (Shaif et al., 2025). The present findings show a significant association between physical activity level and rumination, and rumination was in turn closely associated with sleep quality. From a cognitive processing perspective, this finding provides additional theoretical support for the relationship between physical activity and sleep quality and suggests that rumination may represent an important psychological node linking behavioral factors and sleep outcomes (Gu et al., 2026).

Second, this study further incorporated emotion regulation strategies into the analytical framework by distinguishing between cognitive reappraisal and expressive suppression, two strategies with different psychological functions. The results showed that these strategies exhibited opposite patterns of association between rumination and sleep quality. This finding provides theoretical support for the multidimensionality of emotion regulation, suggesting that emotion regulation strategies should not be viewed simply as “adaptive” or “maladaptive,” but rather as showing differentiated associations with health outcomes under specific psychological contexts (Gross and John, 2003; Springstein and English, 2023). This contributes to a more refined understanding of the functional differences among emotion regulation strategies and extends related theories to the domain of sleep health.

Third, from an integrative perspective, the proposed “physical activity–rumination–emotion regulation–sleep quality” association model helps bridge previously fragmented research approaches that have examined behavioral, cognitive, and emotional factors in isolation. By systematically examining multiple levels of psychological variables within a single model, this study highlights that sleep quality may reflect the combined influence of multiple psychological processes rather than the effect of a single factor.

It should be emphasized that this study is based on cross-sectional data, and the proposed chain mediation model reflects statistical association structures among variables rather than strict causal mechanisms. Nevertheless, within this limitation, the study provides an informative analytical framework for future theoretical development and empirical research. Future studies may build on this framework by using longitudinal designs or experimental methods to further examine the directionality and dynamic changes of different psychological pathways, thereby refining theoretical explanations of the relationship between physical activity and sleep quality.

### Practical implications

From a practical perspective, this study provides useful implications for sleep health promotion and psychological support in university settings. Although the cross-sectional design of the study does not allow for direct causal inference, the association structure identified among the variables helps to clarify the behavioral, cognitive, and emotional characteristics that may jointly relate to sleep problems among college students. These findings offer directional guidance for university health services and psychological support practices.

First, the results suggest that physical activity level should be considered within a comprehensive framework for assessing sleep health among college students. The stable association between physical activity level and sleep quality indicates that physical activity is not only a marker of physical fitness or lifestyle, but may also reflect an individual’s overall daily rhythm. In practice, universities may use students’ participation in physical activity as an auxiliary behavioral indicator for identifying potential sleep risk. Encouraging regular and moderate physical activity may help students establish more stable daily routines.

Second, the findings highlight the important association between rumination and sleep problems among college students. Students with higher levels of rumination tended to report more pronounced sleep difficulties, pointing to a key focus for university mental health services. In counseling and support settings, practitioners may benefit from attending not only to external factors such as schedules and academic demands, but also to students’ cognitive patterns before sleep or under stress, particularly the tendency to repeatedly focus on negative content. Addressing these patterns may allow for more targeted cognitive support for sleep-related difficulties.

Third, the results provide specific implications for emotion regulation–oriented mental health education. The differentiated associations between emotion regulation strategies and sleep quality suggest that universities should emphasize the diversity and contextual suitability of emotion regulation strategies in mental health courses, group counseling, or psychological skills training. Helping students understand the potential differences among regulation strategies in terms of psychological burden and long-term adjustment may be more beneficial than focusing solely on emotional control or suppression.

From an integrated application perspective, the “physical activity–rumination–emotion regulation–sleep quality” framework identified in this study offers a theoretical reference for multi-level and cross-departmental health promotion efforts in university settings. Physical education curricula, extracurricular exercise programs, and mental health education initiatives may be coordinated at the goal level. By combining physical activity promotion with the development of cognitive and emotion regulation skills, universities may provide more systematic and integrated support for students’ sleep health.

Overall, the practical significance of this study does not lie in proposing specific intervention programs or evaluating intervention effects. Rather, by clarifying the association structure among multiple psychological and behavioral variables, the study provides an analytical perspective that may inform sleep health promotion, psychological risk identification, and health education design in university contexts. Future research may build on these findings by incorporating longitudinal or intervention designs to further examine the applicability and effectiveness of different practical approaches.

## Limitations and future directions

Although this study has certain strengths in terms of sample size and variable integration, several limitations should be noted and addressed in future research.

First, this study employed a cross-sectional design, with all variables measured at a single time point. Therefore, it does not allow for strict causal inferences regarding the relationships among physical activity, rumination, emotion regulation strategies, and sleep quality. Although the findings support the statistical plausibility of the proposed chain mediation model, the directionality of the associations among variables requires further verification through longitudinal or experimental research. Future studies may adopt multi-wave longitudinal designs to examine whether changes in physical activity are accompanied by dynamic changes in rumination, emotion regulation strategies, and sleep quality, thereby providing clearer evidence regarding temporal relationships among these variables. Second, this study relied primarily on self-report questionnaires to assess physical activity, rumination, emotion regulation strategies, and sleep quality. As a result, the findings may be influenced by common method bias and social desirability effects. Although statistical tests were conducted to assess common method bias, potential biases associated with self-report measures cannot be fully ruled out. Future research may benefit from incorporating objective measures, such as wearable device–based assessments of physical activity and sleep, or experimental tasks and behavioral indicators to assess cognitive and emotion regulation processes, in order to enhance the objectivity and interpretability of the findings. Third, the sample consisted of college students, with a relatively homogeneous source, which may limit the generalizability of the findings. College students represent a specific population with unique characteristics in terms of daily routines, developmental stage, and sources of stress. Whether the observed association patterns apply to other age groups or occupational populations remains to be determined. Future studies may replicate the proposed model in diverse populations to compare similarities and differences in the relationships between physical activity and sleep quality across developmental stages and social roles.

In addition, although sex and age were controlled, several potentially important confounding variables were not included in the present study, such as depressive symptoms, anxiety, caffeine intake, chronotype, and bedtime regularity. These factors are known to be associated with both physical activity and sleep quality and may therefore have influenced the magnitude of the observed associations. For example, individuals with higher levels of depressive or anxiety symptoms may be less physically active, more prone to rumination, and more likely to report poorer sleep quality, which may have contributed to the magnitude of the observed indirect associations. Similarly, irregular sleep schedules, evening chronotype, or higher caffeine intake may independently contribute to poorer sleep quality, potentially overlapping with the pathways identified in the present model. Therefore, the findings of this study should be interpreted with caution. Future research is needed to incorporate a broader range of psychological and behavioral variables to further examine the robustness and generalizability of the proposed model. Despite these limitations, the relatively large sample size and the consistency of the observed associations across analyses provide support for the robustness of the findings.

In addition, the measure of physical activity used in this study focused on overall activity level and did not capture the timing or type of exercise. As prior research suggests that the sleep-related associations of physical activity may vary according to exercise modality and time of day, especially for vigorous activity performed late in the evening, future studies should incorporate more detailed assessments of physical activity patterns.

Moreover, the present study did not assess or exclude pre-existing clinically diagnosed sleep disorders. It is therefore possible that some participants had significant sleep pathology, which may have influenced the observed associations. Future studies should include screening for clinical sleep disorders and consider this factor in sampling or statistical adjustment.

Overall, this study integrates physical activity, rumination, and emotion regulation strategies to provide an informative framework for understanding sleep quality among college students. With further methodological refinement and the incorporation of more diverse measurement approaches, future research may build on this framework to advance theoretical understanding and provide more rigorous empirical evidence for sleep health promotion.

## Conclusion

This study examined the associations between physical activity and sleep quality among college students and explored the roles of rumination and emotion regulation strategies within this relationship. The findings indicate that physical activity level is associated with sleep quality both directly and indirectly through rumination and emotion regulation strategies. Specifically, rumination and the differential use of cognitive reappraisal and expressive suppression were linked to variations in sleep quality, forming a chain of psychological associations.

By integrating behavioral, cognitive, and emotional factors within a single analytical framework, this study provides a more comprehensive perspective on sleep problems among college students. Although causal conclusions cannot be drawn due to the cross-sectional design, the results highlight the importance of considering cognitive processing and emotion regulation when examining the relationship between physical activity and sleep quality. These findings offer a useful reference for future research and for efforts aimed at promoting sleep and mental health in university settings.

## Data Availability

The original contributions presented in this study are included in the article/Supplementary material, further inquiries can be directed to the corresponding authors.

## Footnotes

1
https://www.wjx.cn/
