# Supplementary material for: Associations between physical activity and sleep quality among college students: the chain-mediating roles of rumination and emotion regulation strategies
Source: Front Psychol. 2026 May 18;17:1835863. doi: 10.3389/fpsyg.2026.1835863 (PMC13222782; doi:10.3389/fpsyg.2026.1835863)
Supplement: Supplementary file 1 [file Table_1.docx]

Table S1. Correlations Among Rumination Subdimensions and Main Variables

| **Variable** | **Symptom Rumination** | **Reflective Pondering** | **Brooding** |
| --- | --- | --- | --- |
| Physical Activity | -0.305** | -0.247** | -0.268** |
| Cognitive Reappraisal | -0.402** | -0.341** | -0.353** |
| Expressive Suppression | 0.312** | 0.299** | 0.296** |
| Sleep Quality (PSQI) | 0.405** | 0.315** | 0.336** |
| Note. *p* < .05, **p** < .01. | | | |
